# Survival prediction by Bayesian network modeling for pseudomyxoma peritonei after cytoreductive surgery plus hyperthermic intraperitoneal chemotherapy

**DOI:** 10.1002/cam4.5138

**Published:** 2022-08-21

**Authors:** Xin Zhao, Xinbao Li, Yulin Lin, Ru Ma, Ying Zhang, Dazhao Xu, Yan Li

**Affiliations:** ^1^ Department of Peritoneal Cancer Surgery Beijing Shijitan Hospital, Peking University Ninth School of Clinical Medicine Beijing China; ^2^ Department of Peritoneal Cancer Surgery Beijing Shijitan Hospital, Capital Medical University Beijing China

**Keywords:** Bayesian network, cytoreductive surgery, hyperthermic intraperitoneal chemotherapy, pseudomyxoma peritonei, survival prognostic model

## Abstract

**Objectives:**

To establish a survival prognostic model for pseudomyxoma peritonei (PMP) treated with cytoreductive surgery (CRS) plus hyperthermic intraperitoneal chemotherapy (HIPEC) based on Bayesian network (BN).

**Methods:**

453 PMP patients were included from the database at our center. The dataset was divided into a training set to establish BN model and a testing set to perform internal validation at a ratio of 8:2. From the training set, univariate and multivariate analyses were performed to identify independent prognostic factors for BN model construction. The confusion matrix, receiver operating characteristic (ROC) curve and the area under curve (AUC) were used to evaluate the performance of the BN model.

**Results:**

The univariate and multivariate analyses identified 7 independent prognostic factors: gender, previous operation history, histological grading, lymphatic metastasis, peritoneal cancer index, completeness of cytoreduction and splenectomy (all *p* < 0.05). Based on independent factors, the BN model of training set was established. After internal validation, the accuracy and AUC of the BN model were 70.3% and 73.5%, respectively.

**Conclusion:**

The BN model provides a reasonable level of predictive performance for PMP patients undergoing CRS + HIPEC.

## Graphical abstract

This study developed a Bayesian network (BN) survival prognostic model for PMP which has a resonable level of predictive performation. The BN model can help oncologists to make clinical decisions.

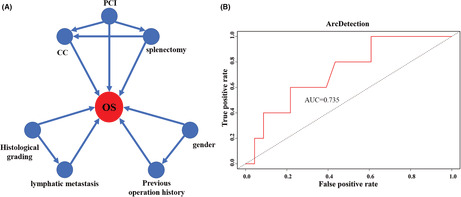

## 1 INTRODUCTION

Pseudomyxoma peritonei (PMP) is a rare malignant tumor and has an estimated incidence of 1–3 per million people annually.^1^, ^2^ Through nearly four decades of clinical research, cytoreductive surgery (CRS) plus hyperthermic intraperitoneal chemotherapy (HIPEC) has been developed and become the normative treatment for PMP patients.^3^, ^4^, ^5^

PMP mainly originates from appendiceal mucinous tumors. Tumor cells and mucus enter the abdominal and pelvic cavity through the perforated appendix wall, accumulate and redistribute in the abdomen and pelvis, leading to mucinous ascites, peritoneal implantation, omentum cake, and organ involvement particularly to the ovary and the spleen.^1^, ^2^, ^3^, ^4^

There are many factors affecting the prognosis of PMP, such as age, peritoneal cancer index (PCI), completeness of cytoreduction (CC), histological grading, lymphatic metastasis, vascular invasion, stripped peritoneum area, number of anastomosis.^6^, ^7^, ^8^ The identification of prognostic factors and development of survival prognostic model for PMP are important to predict the clinical outcome for PMP patients treated with CRS + HIPEC and to make clinical treatment decision.

In recent years, machine learning method has been widely used in medical field.^9^ Bayesian network (BN) is a directed acyclic graph that explores the unknown probability of variables from the known probability knowledge. Previous studies have developed BN model to survival prediction of malignant tumors such as lung cancer, breast cancer, gallbladder cancer and colon cancer,^10^, ^11^, ^12^, ^13^ which showed a high forecast accuracy. At present, there is no research on the establishment of PMP survival prognostic model based on BN. Therefore, this study aims to construct and evaluate a BN prediction model for PMP.

## 2 MATERIALS AND METHODS

### 2.1 Clinical information

Our institute is a medical center specialized in treating peritoneal metastases from gastrointestinal and gynecological malignancies, mainly using the CRS + HIPEC and postoperative integrated treatment approaches. Each patient treated at our center has been entered into a prospectively established database, which contained detailed clinicopathological information on 1980+ patients. From this database, we screened 453 PMP patients underwent CRS + HIPEC for the first time from December 2004 to July 2021. All patients met the following inclusion and exclusion criteria.^14^ The study was approved by the Ethics Committee of Beijing Shijitan Hospital. All patients signed the informed consent.

Major inclusion criteria were: (1) pathological diagnosis of PMP; (2) Karnofsky performance status (KPS) score > 60; (3) white blood cells ≥3.5 × 10^9^/L and platelet ≥80 × 10^9^/L; (4) serum bilirubin, aspartic aminotransferase and alanine aminotransferase <2 × the upper limit of normal (ULN); (5) serum creatinine <1.2 × ULN; and (6) cardiac and pulmonary functions can stand major operation.

Major exclusion criteria were: (1) lung, brain, bone or liver metastases; (2) serum bilirubin, aspartic aminotransferase and alanine aminotransferase ≥2 × ULN; (3) serum creatinine ≥1.2 × ULN; (4) severe mesenteric contracture; and (5) major organ functions cannot stand major operation.

### 2.2 CRS + HIPEC

After general anesthesia, a midline xiphoid‐pubic incision was performed to enter the abdomen. Once the abdominal wall was opened, characteristics and volume of ascites were recorded and evaluation of PCI was conducted, according to Sugarbaker's principle.^15^ Then, the maximal CRS was performed, including the resection of the visceral and parietal peritoneum, tumor‐involved organs, and lymphadenectomy.

CC score was evaluated after CRS according to Sugarbaker's criteria.^15^ CC0, no residual peritoneal disease after CRS; CC1, residual tumor <0.25 cm; CC2, residual tumor 0.25–2.5 cm; and CC3, residual tumor >2.5 cm or the presence of unresectable tumor nodules.

After CRS, open HIPEC was performed. The chemotherapy drugs were docetaxel 120 mg + cisplatin 120 mg or cisplatin 120 mg + mitomycin C 30 mg, each dissolved in 3000 ml of heated saline at 43°C for 60 min.

Then, digestive tract and urinary tract reconstructions were performed after HIPEC. Intestinal stoma was conducted if necessary. Drainage tubes were placed and the incision was sutured with reduced tension. After operation, patient was delivered to the intensive care unit for recovery and transferred to the surgical oncology ward when the condition stabilized.

### 2.3 Follow‐up

The follow‐up consisted of physical examination, tumor response evaluation and survival information. The frequency of follow‐up was once every 3 months within 2 years after CRS + HIPEC, once every 6 months for the third year after CRS + HIPEC and once every year thereafter.^16^ The last follow‐up was on December 31, 2021, with the rate of 100%.

### 2.4 Definition

Overall survival (OS): OS was defined as the time interval from the date of clinical diagnosis to the date of death or the last follow‐up.

### 2.5 Statistical analysis

SPSS 26.0 (IBM Corporation, SPSS, Armonk, NY) were used for data collection and analysis. Continuous variables were reported as median (range) and compared with *t*‐test or rank sum test. Categorical variables were presented as number (percentage), analyzed by *x* ^2^ test and Fisher's exact method. Kaplan–Meier method was used to estimated OS and log‐rank test was used for comparison between groups. *p* value <0.05 was considered significant. Univariate and multivariate COX regression analyses were conducted to identify the independent risk factors on OS. R software (version 4.1.2 developed by The R Foundation for Statistical Computing) was used for BN model development and evaluation.

### 2.6 Development of the BN model

The “*Bnlearn*” package (version 4.7) was used for BN structure learning, parameter learning and inference. To evaluate the BN model performance, all PMP patients were randomly split in training set and testing set with a ratio of 8:2. The training set was used to establish the BN model and the testing set performed internal validation. From the training set, univariate and multivariate analyses were performed to screen for independent prognostic factors for BN model construction. We selected OS as the target variable and 36 months as the target cut‐off point time. As the “*Bnlearn*” package can only deal with discrete variables, discretization of the data was completed prior to the construction of the model. After establishment of the dataset and discretization of variables into discrete variables, a BN model was established.

### 2.7 Evaluation of BN model

The confusion matrix is a cross table containing the observed and predicted classes with relevant statistics, which can be obtained by internal validation. The accuracy of the BN model is defined by the following equation: Accuracy = [true positive (TP) + true negative (TN)]/[TP + false positive (FP) + TN + false negative (FN)]. Using the “*ROCR”* package (version 1.0–11), the receiver operating characteristic curve (ROC) and the area under curve (AUC) were calculated to evaluate the overall performance of the BN model.

## 3 RESULTS

### 3.1 Major clinicopathological characteristics comparison between the training set and testing set

A total of 453 PMP patients undergoing CRS + HIPEC between 2004 and 2021 were included. There were 207 (45.7%) males and 246 (54.3%) females for the whole cohort. Patients ranged in age from 24 to 81 years (median 55). In terms of histological grading, there were 248 (54.7%) cases with low grade, 158 (34.9%) cases with high grade, and 47 (10.4%) cases with high grade with signet ring cells. The median duration of CRS + HIPEC was 630 min (range: 95–1080 min). The median number of resected organs and peritoneum were 3 (0–10) and 5 (0–9), respectively. The median PCI was 30 (range:1–39). There were 227 (50.1%) cases with CC0‐1 and 226 (49.9%) cases with CC2‐3.

There were 362 (80.0%) patients in the training set and 91 (20.0%) patients in the testing set. The baseline characteristics of the training set and testing set were balanced, and there were no statistically significant differences in clinicopathological characteristics of two sets (all *p* > 0.05) (Table 1).

**TABLE 1:** Baseline characteristics for the training set and testing set

Variable	Training set (*n*1 = 362)	Testing set (*n*2 = 91)	*p* value
Gender, *n* (%)			0.399
Male	169 (46.7)	38 (41.8)	
Female	193 (53.3)	53 (58.2)	
Age (years), median (range)	55 (26–81)	56 (24–76)	0.392
BMI (kg/m^2^), median (range)	23.0 (15.2–40.0)	22.5 (16.3–31.9)	0.583
Previous operation history, *n* (%)			0.649
No	96 (26.5)	22 (24.2)	
Yes	266 (73.5)	69 (75.8)	
Chemotherapy history, *n* (%)			0.569
No	199 (55.0)	47 (51.6)	
Yes	163 (45.0)	44 (48.4)	
KPS score, median (range)	90 (60–100)	90 (60–100)	0.467
Histological grading, *n* (%)			0.949
Low grade	199 (55.0)	49 (53.8)	
High grade	125 (34.5)	33 (36.3)	
High grade with signet ring cells	38 (10.5)	9 (9.9)	
Vascular invasion, *n* (%)			0.992
No	346 (95.6)	87 (95.6)	
Yes	16 (4.4)	4 (4.4)	
Lymphatic metastasis, *n* (%)			0.201
No	337 (93.1)	88 (96.7)	
Yes	25 (6.9)	3 (3.3)	
Operative duration (min), median (range)	629.5 (95–1065)	635.0 (120–1080)	0.763
Resected organs, median (range)	3 (0–10)	3 (0–8)	0.534
Stripped peritoneum area, median (range)	6 (0–9)	5 (0–9)	0.113
Splenectomy			0.068
No	223 (61.6)	68 (74.7)	
Yes	139 (38.4)	23 (25.3)	
Anastomosis, *n* (%)			0.314
No	111 (30.7)	23 (25.3)	
Yes	251 (69.3)	68 (74.7)	
PCI, median (range)	30 (1–39)	31 (1–39)	0.348
CC, *n* (%)			0.542
0–1	184 (50.8)	43 (47.3)	
2–3	178 (49.2)	48 (52.7)	
RBC transfusion volume (U), median (range)	2 (0–20)	4 (0–14)	0.110
Plasma transfusion volume (ml), median (range)	800 (0–2000)	800 (0–1600)	0.365
Fluid transfusion volume (ml), median (range)	6800 (1000–102,500)	6500 (2000–17,530)	0.831
Blood loss volume (ml), median (range)	600 (50–5000)	600 (100–4800)	0.892
Ascites volume (ml), median (range)	600 (0–20,000)	1000 (0–20,000)	0.260

Abbreviations: BMI, body mass index; CC, completeness of cytoreduction; KPS, Karnofsky performance status; PCI, peritoneal cancer index; RBC, red blood cell.

### 3.2 BN model construction by training set

**FIGURE 1:**
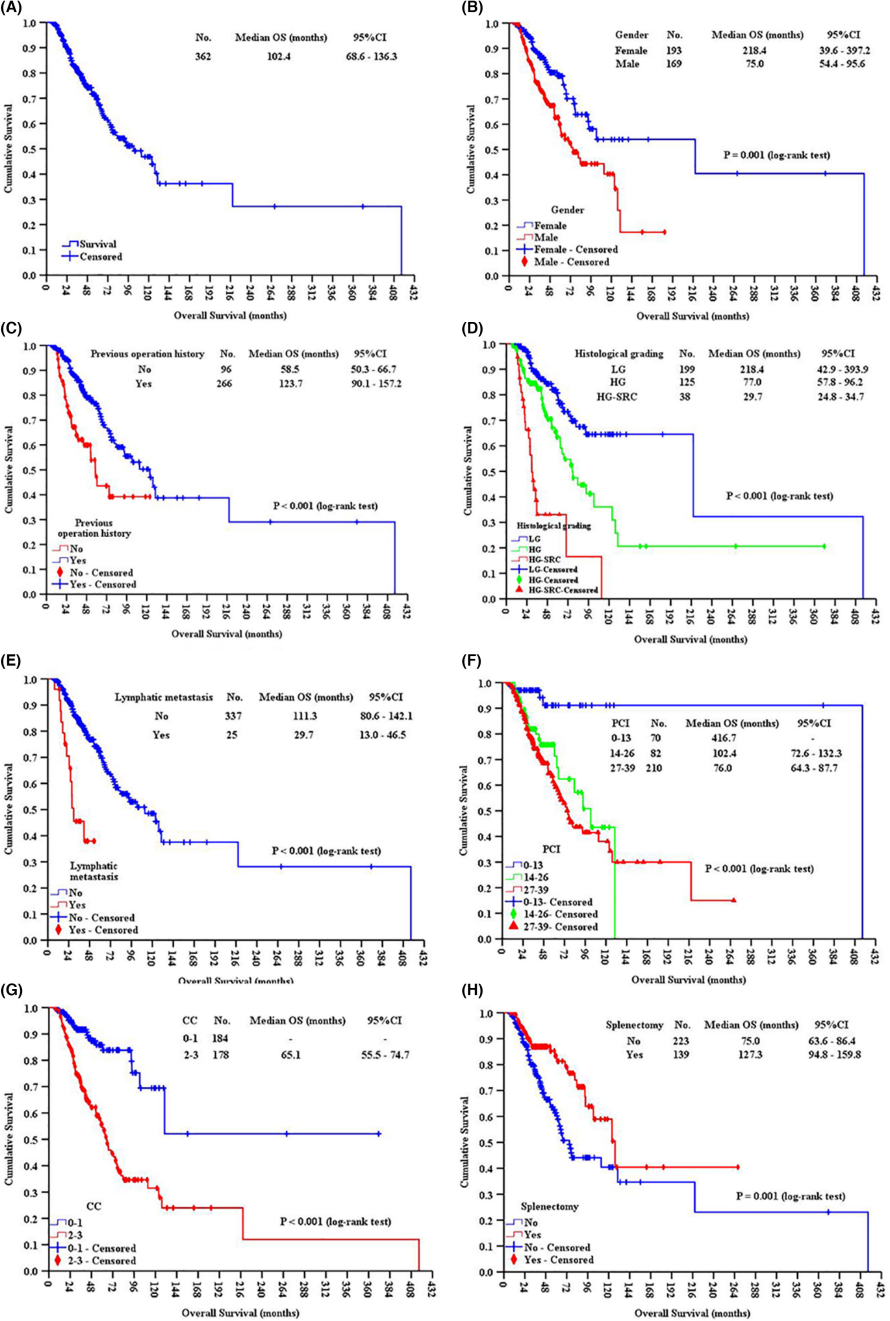
Kaplan–Meier curves of training set (A), and subgroup comparation based on gender (B), previous operation history (C), histological grading (D), lymphatic metastasis (E), PCI (F), CC (G), and splenectomy (H). HG, high grade; HG‐SRC, high grade with signet ring cells; LG, low grade.

**FIGURE 2:**
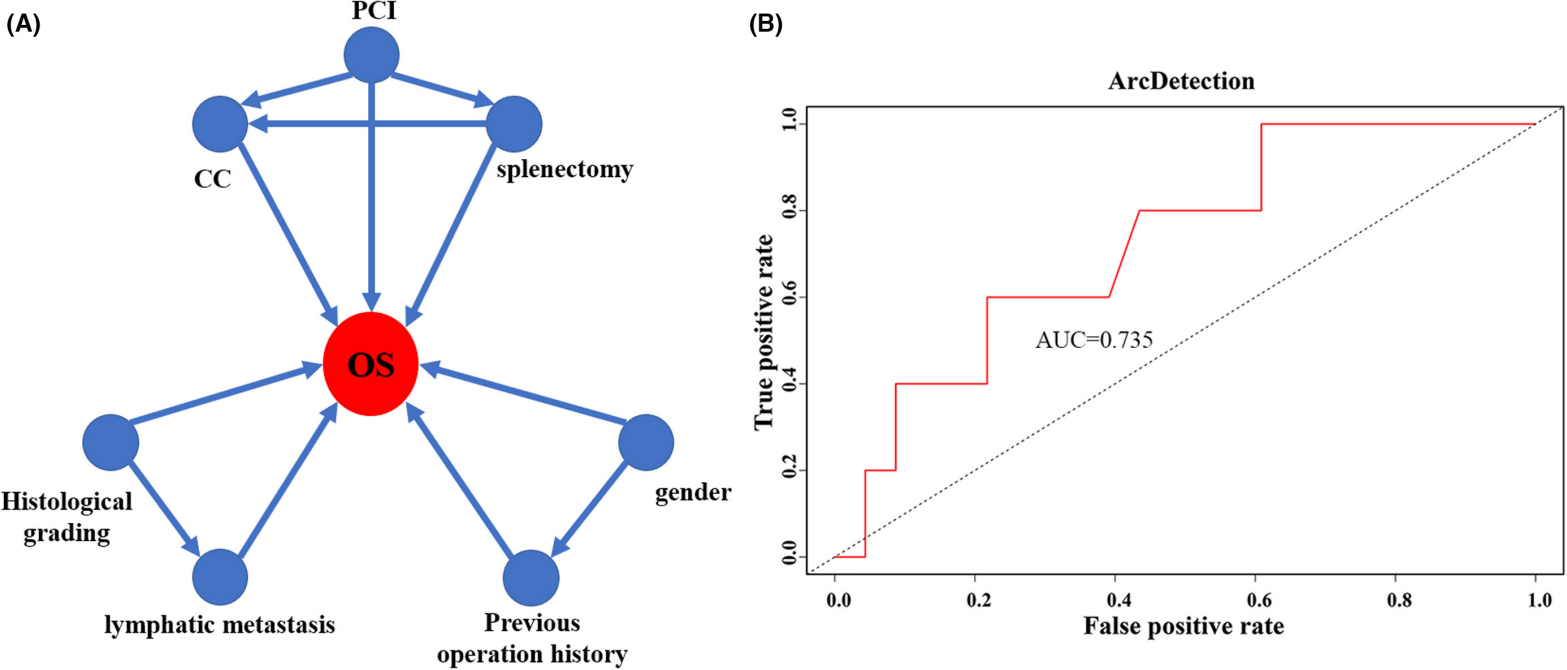
Construction and ROC validation of the BN model. (A) The BN model shows the interactions of the 7 independent factors and their combined contribution to OS, with a prediction accuracy of 70.3%; (B) ROC analysis for internal validation shows the AUC of this BN model being 73.5%.

Univariate analysis revealed the following 14 factors having significant impact on mOS: gender, BMI, previous operation history, chemotherapy history, histological grading, vascular invasion, lymphatic metastasis, PCI, CC, number of organ resections, number of anastomoses, RBC transfusion volume, ascites volume, and splenectomy (all *p* < 0.05) (Table 2). Factors with *p* < 0.05 were incorporated into multivariate COX regression analysis, which identified 7 independent prognostic factors: gender, previous operation history, histological grading, lymphatic metastasis, PCI, CC and splenectomy (all *p* < 0.05) (Table 3). Kaplan–Meier curves of training set and subgroup comparation based on those 7 independent prognostic factors are showed in Figure 1A–H. Based on the 7 independent prognostic factors above, the BN model for training set was constructed (Figure 2A).

**TABLE 2:** Univariate survival analysis on training set

Items	No (%)	mOS (95%CI) (months)	*p* value
Gender			<0.001
Male	169 (46.7)	75.0 (54.4–95.6)	
Female	193 (53.3)	218.4 (39.6–397.2)	
Age (years)			0.264
<65	302 (83.4)	102.4 (68.6–136.3)	
≥65	60 (16.6)	77.7 (24.6–130.7)	
BMI (kg/m^2^)			0.012
<25	268 (74.0)	92.8 (60.9–124.7)	
≥25	94 (26.0)	102.4 (−)	
Previous operation history			<0.001
No	96 (26.5)	58.5 (50.3–66.7)	
Yes	266 (73.5)	123.7 (90.1–157.2)	
Chemotherapy history			0.002
No	199 (55.0)	130.4 (26.1–234.6)	
Yes	163 (45.0)	70.0 (50.0–90.0)	
Histological grading			<0.001
Low‐grade	199 (55.0)	218.4 (42.9–393.9)	
High‐grade	125 (34.5)	77.0 (57.8–96.2)	
High‐grade with signet ring cells	38 (10.5)	29.7 (24.8–34.7)	
Vascular invasion			<0.001
No	346 (95.6)	102.4 (68.7–136.1)	
Yes	16 (4.4)	30.8 (23.4–38.2)	
Lymphatic metastasis			<0.001
No	337 (93.1)	111.3 (80.6–142.1)	
Yes	25 (6.9)	29.7 (13.0–46.5)	
PCI			<0.001
0–13	70 (19.3)	416.7 (−)	
14–26	82 (22.7)	102.4 (72.6–132.3)	
27–39	210 (58.0)	76.0 (64.3–87.7)	
CC			<0.001
0–1	184 (50.8)	‐	
2–3	178 (49.2)	65.1 (55.5–74.7)	
Resected organs			0.012
≤2	145 (40.1)	75.0 (45.3–104.7)	
>2	217 (59.9)	416.7 (−)	
Stripped peritoneum area			0.521
≤5	177 (48.9)	102.4 (52.0–152.9)	
>5	185 (51.1)	93.4 (64.6–122.1)	
Number of anastomoses			0.021
0	111 (30.7)	66.9 (49.7–84.2)	
≥1	251 (69.3)	127.3 (78.7–175.9)	
RBC transfusion volume (U)			0.032
<5	274 (77.0)	130.4 (60.4–200.3)	
≥5	82 (23.0)	70.0 (49.9–90.1)	
Plasma transfusion volume (ml)			0.597
<800	166 (46.6)	111.3 (72.7–150.0)	
≥800	190 (53.4)	93.4 (5.8–180.9)	
Fluid transfusion volume (ml)			0.075
<5000	74 (20.9)	67.1 (15.0–119.3)	
≥5000	280 (79.1)	111.3 (81.6–141.1)	
Blood loss volume (ml)			0.179
<800	206 (56.9)	130.4 (47.0–213.7)	
≥800	156 (43.1)	77.7 (56.7–98.7)	
Ascites volume (ml)			<0.001
<1000	192 (53.3)	127.3 (91.6–163.0)	
≥1000	168 (46.7)	70.0 (58.5–81.6)	
Splenectomy			0.001
No	223 (61.6)	75.0 (63.6–86.4)	
Yes	139 (38.4)	127.3 (94.8–159.8)	

Abbreviations: BMI, body mass index; CC, completeness of cytoreduction; CI, confidence interval; mOS, median overall survival; PCI, peritoneal cancer index; RBC, red blood cell.

**TABLE 3:** Multivariate COX regression analysis for independent prognostic factors

Items	Wald	HR	95%CI	*p*
Gender (male vs. female)	7.065	2.348	2.052–2.596	<0.001
Previous operation history (yes vs. no)	19.969	0.320	0.194–0.527	<0.001
Histological grading	19.775			<0.001
High‐grade versus Low‐grade	6.470	1.820	1.147–2.888	0.011
High‐grade with signet ring cells versus high‐grade	19.525	3.849	2.117–6.999	<0.001
Lymphatic metastasis (yes vs. no)	9.623	2.896	1.479–5.669	0.002
PCI	8.471			0.004
14–26 versus 0–13	8.391	6.618	1.843–23.773	0.004
27–39 versus 14–26	6.063	4.774	1.376–16.567	0.014
CC (2–3 vs. 0–1)	9.029	2.385	1.353–4.204	0.003
Splenectomy (yes vs. no)	19.352	0.353	0.222–0.561	<0.001

Abbreviations: CC, completeness of cytoreduction; CI, confidence interval; HR, hazard ratio; PCI, peritoneal cancer index.

### 3.3 Internal validation for BN model

The confusion matrix of internal validation is listed in Table 4. In testing set, there were 37 patients who with survival ≤36 months and 54 patients with survival >36 months. A total of 22 patients were correctly classified as having survival ≤36 months and 42 patients were classified as having survival >36 months. The accuracy of the model was 70.3%. ROC curve for the BN model developed on the testing set and the area under the curve (AUC) was 73.5% for the BN model (Figure 2B).

**TABLE 4:** Confusion matrix of BN model

Predicted	Reference		Total (*n*)
	≤36 months (*n*)	>36 months (*n*)	
≤36 months (*n*)	22	12	34
>36 months (*n*)	15	42	57
Total (*n*)	37	54	91

## 4 DISCUSSIONS

The development and utilization of cancer survival prediction models are of great significance for physicians to make clinical decisions. In this study, we constructed a BN model to predict survival of PMP patients based on the 7 independent prognostic factors. After internal validation, the BN model showed a reasonable level of predictive performance with the accuracy being 70.3% and the AUC being 73.5%.

The univariate and multivariate analyses of training set showed that gender, previous operation history, histological grading, lymphatic metastasis, PCI, CC and splenectomy were the independent prognostic factors. Chua et al.^17^ conducted a large multi‐center study of 2298 patients, which showed age, severe adverse events, CC and PMP with high grade were independent risk factors for OS. Another study conducted by Ansari et al.^18^ have confirmed that male, high grade PMP, high level of carbohydrate antigen (CA) 125 and carcinoma embryonic antigen (CEA) were independent risk factors for poor prognosis. As mentioned above, there are many factors affecting the prognosis of PMP, and there are certain differences among PMP cases in different treatment centers.

Among 7 independent prognostic factors selected by multivariate analysis for our study, there were two factors associated with CRS + HIPEC, which were splenectomy and CC score. Our study showed that splenectomy provided a significantly better survival comparing with non‐splenectomy for PMP patients. The reason may be that splenectomy enhances the likelihood of complete cytoreduction. However, a study^19^ showed that splenectomy could increase major complication rate in patients with CRS + HIPEC. So, the efficacy and perioperative safety of splenectomy need further study to verify. CC score is a critical independent prognostic factor for PMP patients. As shown in the BN model we constructed, PCI and splenectomy have big impacts on CC score. PMP patients with low PCI and splenectomy, underwent standardized CRS + HIPEC, had a lower CC score and a longer OS. Histological grading and lymphatic metastasis are also independent factors affecting the survival and prognosis of PMP patients. The BN model showed that histological grading was correlated with lymphatic metastasis, and the lymphatic metastasis rate was higher in patients with high pathological grade.

In 2001, Sugarbaker systematically studied CRS + HIPEC+ early postoperative intraperitoneal chemotherapy (EPIC) for PMP, demonstrating that this therapy was the optimal treatment strategy for PMP patients. This treatment embodies the advantages of comprehensive treatment based on surgery, integrating the synergistic effects of surgical resection, regional chemotherapy, hyperthermia and large volume liquid lavage. CRS can remove all visible tumor tissues and HIPEC can eliminate micro‐metastases and free tumor cells. Current studies^17^, ^18^, ^20^, ^21^, ^22^, ^23^, ^24^, ^25^ have reported that the mOS of PMP treated with standard CRS + HIPEC was 103.4–196.0 months, the median progression‐free survival time was 40.0–98.0 months, and the 5‐ and 10‐year survival rates were 49.0%–92.1% and 32.8%–80.8%, respectively. The mOS of the training set in this study was 102.4 months, and the 3‐, 5‐ and 10‐year survival rates were 82.3%, 68.1% and 43.9%, respectively. One early study of our center^26^ showed that the mOS of 254 PMP patients was 55.4 months, and 3‐ and 5‐year survival rates were 61.0% and 44.3%, respectively. CRS + HIPEC can prolong the survival time of PMP obviously.

Currently, HIPEC regimens vary in different treatment centers. Oxaliplatin and mitomycin C are the most commonly basic chemotherapy drugs for HIPEC. There is no international consensus on the best drug and dose for HIPEC. Therefore, international peritoneal cancer centers need to strengthen cooperation and conduct multi‐center, large sample randomized controlled clinical trials to explore HIPEC protocol with high efficacy and less toxicity.

The nomogram is a graphical representation that has been used to predict cancer survival in recent years. Two studies^27^, ^28^ had developed nomograms for predicting survival in PMP patients. Chen et al.^27^ performed a nomogram to predict OS incorporated with age, grade, location, T stage, N stage, M stage, lymph node removed and chemotherapy. The C‐index of the nomogram model was 0.757 after the analysis of the internal validation. Another nomogram survival model proposed by Bai et al.^28^ was based on 5 independent prognostic factors, which were D‐dimer level, carbohydrate antigen (CA) 125 level, CA19‐9 level, degree of radical surgery and histological grade. The C‐index of the model was 0.825 and they did not mention the AUC of the model. Nomogram and BN model both based on the independent risk factors. BN model can further illuminate the relationships and interactions among the independent factors. Moreover, BN model is a direct and structured illustration of how the factors working together to contribute to the outcome. Researchers can improve accuracy of the model by adjusting the conditional probability of each variable node according to clinical experience and research.

In recent years, BN has been widely used in artificial intelligence, systematic biology, disease diagnosis and prognosis, scientific decision‐making and other fields. The application value of BN in medical field is also prominent. The BN survival prediction model has the following advantages: (1) The model is presented in the form of tree graph, which is simple and intuitive; (2) The correlation between variables can be found and the conditional probability of each variable can be calculated and predicted; (3) The inference function of BN can guide treatment decision‐making.

There were three major deficiencies in this study: (1) The survival prognosis model established in this study was based on single‐center data, and only conducted internal validation without external validation; (2) The time span of the cases included in this study was long, which resulted in heterogeneity of the cases; (3) Preoperative tumor markers, Ki‐67, P53 and other pathological indicators were not included in this study.

For the results of this study, the prediction accuracy of the BN model remains to be further improved. In future study, we will expand the sample size, include more variables and conduct external validation to improve the prediction accuracy of the survival prognostic model of PMP.

## 5 CONCLUSIONS

To conclude, this study established a BN‐based survival prediction model for PMP from 7 independent prognostic factors, which could help clinical treatment decision making and outcome prediction.

## AUTHOR CONTRIBUTIONS

Yan Li contributed to study concept. Yan Li and Xin Zhao contributed to study design. Xin Zhao contributed to literature research. Xin Zhao, Xin‐Bao Li, Yu‐Lin Lin, Ru Ma, Da‐Zhao Xu and Ying Zhang contributed to data acquisition. Xin Zhao and Yan Li contributed to data analysis/interpretation. Xin Zhao contributed to statistical analysis. Xin Zhao contributed to manuscript definition of intellectual content. Xin Zhao and Yan Li contributed to manuscript editing. Yan Li contributed to manuscript revision/review. Yan Li contributed to manuscript approval.

## FUNDING INFORMATION

This work was supported by Natural Science Foundation of China (82073376).

## CONFLICT OF INTEREST

None.

## Data Availability

Not available.
